# Absent Anterior Cruciate Ligament

**DOI:** 10.5334/jbr-btr.862

**Published:** 2015-09-15

**Authors:** S. Vanden Bossche, B. Vanzieleghem, H. Declercq, K. V. Verstraete

**Affiliations:** 1 Department of Radiology, AZ Sint-Blasius, Dendermonde, Belgium; 2 Department of Radiology, Ghent University Hospital, Ghent, Belgium

**Keywords:** Knee, ligaments, menisci

## Abstract

This case report presents the MRI findings of aplasia of the anterior cruciate ligament with associated hypoplasia of the posterior cruciate ligament (Manner type 2). Radiographically the presence of a shallow femoral notch and hypoplastic tibial spines (the so-called “dromedar” sign) can aid in the diagnosis. Operative treatment is often not indicated since the congenital absence of the ACL implies longstanding altered biomechanics to which the knee has well adapted in the majority of cases.

Congenital absence of the anterior cruciate ligament (ACL) is an extremely rare condition (prevalence 0.017 per 1 000 live births), first described by Giorgi in 1956 [1]. It has been described as an isolated finding, in association with extra-articular abnormalities such as congenital short femur and fibular hemimelia as well as in association with intra-articular abnormalities such as absent or hypoplastic menisci and osteochondritis dissecans [2]. We report the case of a young woman who has already undergone a leg lengthening procedure with aplasia of the anterior cruciate ligament and hypoplasia of the posterior cruciate ligament (PCL).

## Case report

### Clinical presentation

A 25-year-old woman was referred to our radiology department by her family doctor for an MRI examination of the left knee because of a “crackling” noise of three months duration. There were no complaints of instability, swelling or pain. Physical examination showed anterior laxity of the knee. There was no recent history of trauma. The patient is known with a congenital shortening of the left leg for which she has already undergone a leg lengthening procedure.

### Imaging findings

**Figure 1:**
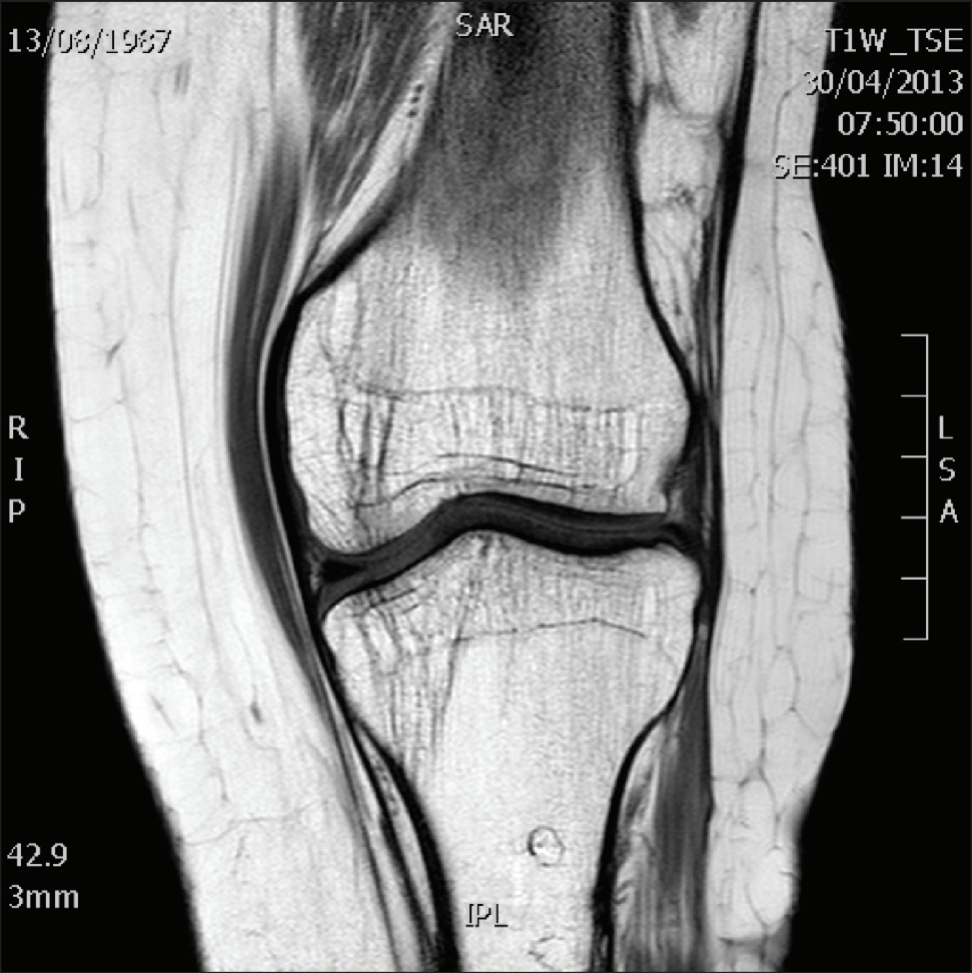
Coronal T1-weighted image of the left knee shows the shallow femoral notch and aplastic lateral tibial spine. The medial tibial spine is present but hypoplastic, also referred to as the “dromedar” sign (arrow). The anterior nor posterior cruciate ligament can be seen in the femoral notch.

**Figure 2:**
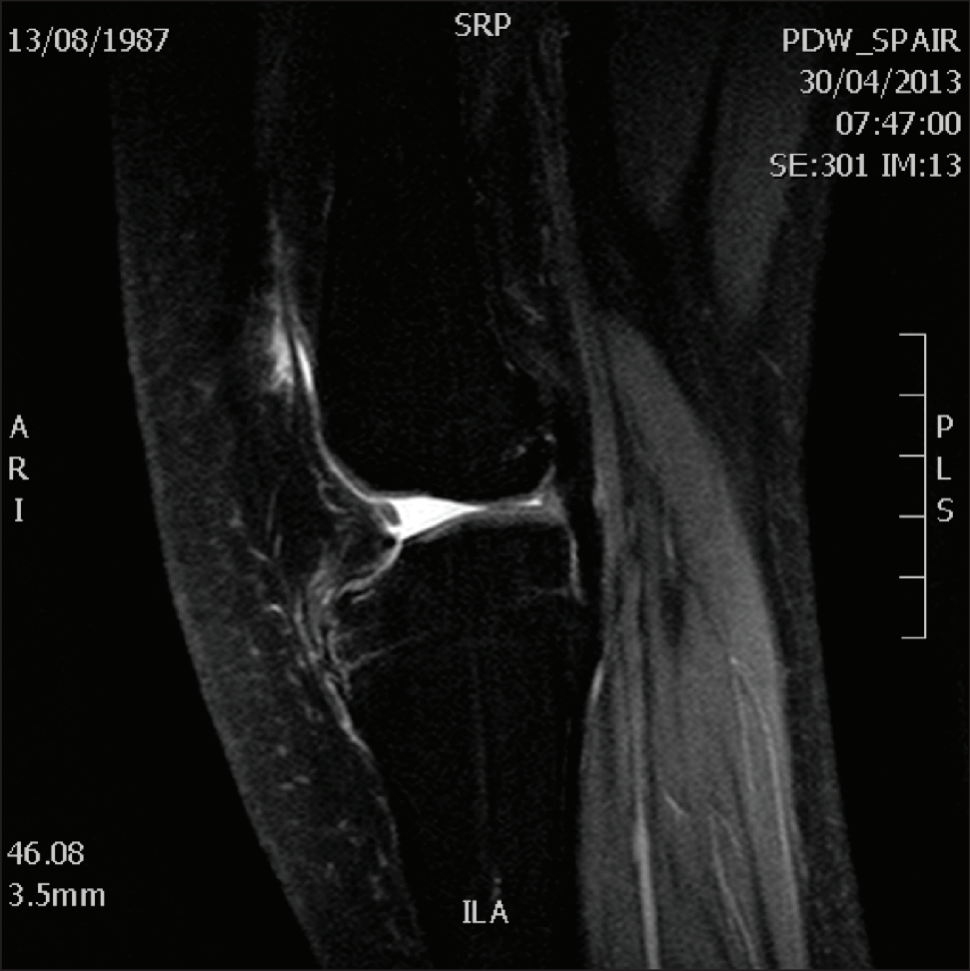
Sagittal fat-suppressed proton density weighted image of the left knee shows the hypoplastic PCL more posteriorly and vertically than expected (arrow).

**Figure 3:**
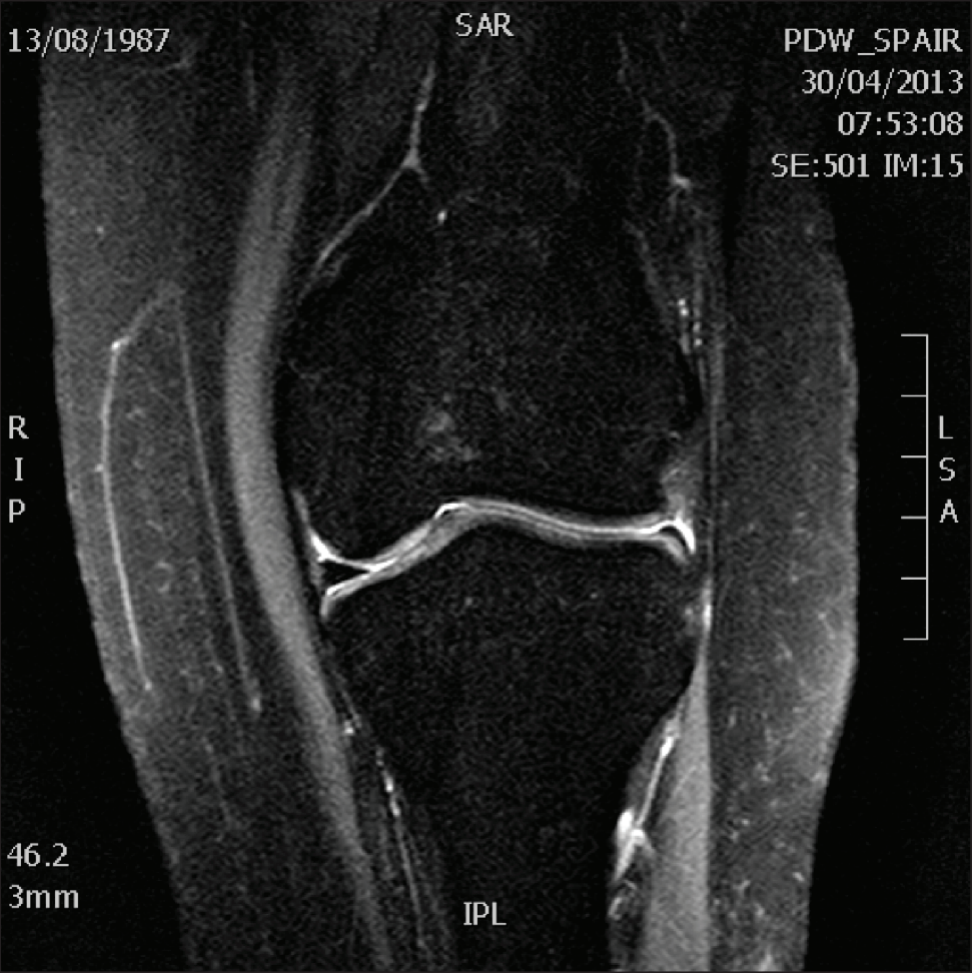
Coronal fat-suppressed proton density weighted image of the left knee shows the hypoplastic lateral meniscus compared to the medial meniscus.

**Figure 4:**
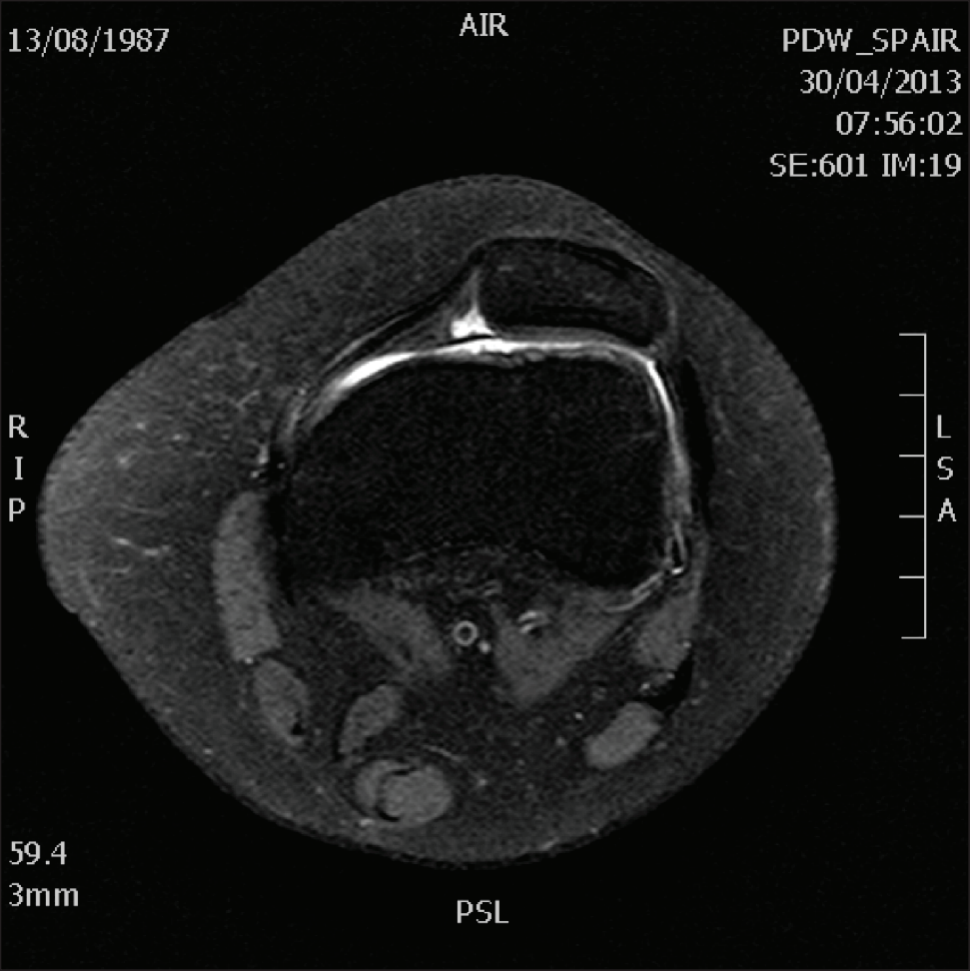
Tranverse fat-suppressed proton density weighted image of the left knee illustrates the hypoplastic lateral femoral condyle (*), hypoplastic medial patellar facet and secondary trochlear dysplasia.

The MRI examination shows multiple anatomic anomalies. The most notable is the absence of the anterior cruciate ligament (Fig. 1). The posterior cruciate ligament is present but appears hypoplastic (Fig. 2). The lateral intercondylar spine is absent and the lateral meniscus is hypoplastic (Fig. 3). There is severe trochlear dysplasia due to hypoplasia of the lateral femoral condyle and medial patellar facet hypoplasia (Fig. 4). Sequellae of earlier leg lengthening procedure can be seen: the left fibula is absent and metallic artefacts are present in the tibia.

### Treatment

Up to this date, the patient was treated conservatively.

## Discussion

Cruciate ligament aplasia is a very rare condition (0.017 per 1000 live births), which might present unilaterally or bilaterally and involves the ACL and PCL in variable extent. Manner et al who conducted the largest study thus far on dysplasia of cruciate ligaments (34 knees in 31 patients) propose a MRI classification based on the appearance of the PCL. Type I represents a hypoplastic (15%) or aplastic ACL (41%) with a normal PCL, type II represents an aplastic ACL with a hypoplastic PCL (type II, 21%) and type III represents an aplastic ACL with an aplastic PCL (type III, 24%). The authors also found that there are radiographic abnormalities, which vary according to the MRI type. Firstly, femoral condylar notch width and depth is decreased according to type: from partial closure of the femoral notch in type I to complete absence in type III. Secondly, the developmental stage of the medial and lateral tibial spine varies according to type: the medial tibial spine is normal in type I, hypoplastic in type II and aplastic in type III aplasia; the lateral tibial spine is hypoplastic or aplastic in type I and aplastic both in type II and type III aplasia. Due to the shallow cartilaginous femoral intercondylar notch and absent tibial intercondylar eminence in type III dysplasia, a true “ball-and-socket” joint is formed by the convex distal femur and concave tibial surface [1].

In our patient, a type II dysplasia of the cruciate ligaments is present, leading to an altered knee joint in which only the lateral femoral intercondylar notch is cartilagenously covered and acts as an accessory joint surface. Other frequent associated findings are meniscal hypoplasia, lateral femoral condyle hypoplasia, patellar hypoplasia and most commonly lower limb longitudinal deficiency, which were all present in our patient [3,4].

It is believed that the cruciate ligaments originate from the interchondral disc – as do the intra-articular structures – and subsequently migrate outside the joint through invagination of the posterior joint capsule. This phased migration theory would explain the co-existence of the intra-articular anomalies in ACL agenesis. It is believed that ACL agenesis is caused by an event occurring in the seventh or eight post-ovulatory week, arresting the development of the cruciate ligaments. The PCL develops before the ACL, which explains why solitary PCL agenesis has not been described. The timing of arrest determines the type of dysplasia: early events are believed to lead to the absence of both ACL and PCL (Manner type III); later events will have allowed formation of PCL to some extent (partially in type II, completely in type I). The absence of the cruciate ligaments will not scallop the femur to form the femoral notch, nor will traction of the cruciate ligaments at the level of the tibia lead to the formation of the tibial spines, explaining these associated findings [5].

There is no consensus on the treatment of cruciate ligament agenesis. Most authors agree on conservative treatment for several reasons. First of all most patients do not complain of instability in spite of the positive clinical tests for ligament insufficiency such as Lachman test and anterior drawer sign. Secondly, the knee as a whole is anomalous and compensatory articular joint surfaces have developed to surmount the defects. Finally, since the femoral condylar notch is narrower in cruciate ligament aplasia, arthroscopic treatment poses significant technical difficulty. The shallow notch and consequently lack of space for graft placement necessitates additional notchplasty or partial condylar resection, which destroys the compensatory cartilaginous changes [4]. A minority considers cruciate dysplasia as a mechanical problem that might lead to instability and believes that ACL reconstruction is indicated, although there are no studies available on long-term results [2].

## Conclusion

This case study illustrates the MRI findings of ACL aplasia with PCL hypoplasia (Manner type 2). Associated anomalies include lateral femoral condyle and patellar hypoplasia with secondary trochlear dysplasia, lateral meniscal hypoplasia and lower limb longitudinal deficiency. A shallow intercondylar notch and hypoplastic to aplastic tibial spines are indicative of the congenital origin and can also be appreciated radiographically. Ligamentoplasty is generally not indicated since there are no subjective signs of instability in the majority of cases, despite the objective indications of ligamentous insufficiency.

## Competing Interests

The authors declare that they have no competing interests.

## References

[1] Manner HM, Radler C, Ganger R, Grill F (2006). Dysplasia of the Cruciate Ligaments: Radiographic Assessment and Classification. J Bone Jt Surg.

[2] Gabos PG, El Rassi G, Pahys J (2005). Knee reconstruction in syndromes with congenital absence of the anterior cruciate ligament. J Pediatr Orthop.

[3] Thomas NP, Jackson AM, Aichroth PM (1985). Congenital absence of the anterior cruciate ligament. A common component of knee dysplasia. J Bone Joint Surg Br.

[4] Balke M, Mueller-Huebenthal J, Shafizadeh S, Liem D, Hoeher J (2010). Unilateral aplasia of both cruciate ligaments. J Orthop Surg Res.

[5] Berruto M, Gala L, Usellini E, Duci D, Marelli B (2012). Congenital absence of the cruciate ligaments. Knee Surg Sports Traumatol Arthrosc.

